# Development of a Radiolabeled Cyclin-Dependent Kinases 4 and 6 (CDK4/6) Inhibitor for Brain and Cancer PET Imaging

**DOI:** 10.3390/ijms25136870

**Published:** 2024-06-22

**Authors:** Chun-Han Huang, Palwasha Khan, Sulan Xu, Jules Cohen, Georgios V. Georgakis, Nashaat Turkman

**Affiliations:** 1 Stony Brook Cancer Center, Stony Brook, Long Island, NY 11794, USA; 2 Department of Radiology, School of Medicine, Stony Brook University, Long Island, NY 11794, USA; 3 Department of Biomedical Engineering, Stony Brook University, Long Island, NY 11794, USA; 4 Department of Medicine, School of Medicine, Stony Brook University, Long Island, NY 11794, USA; 5 Department of Surgery, School of Medicine, Stony Brook University, Long Island, NY 11794, USA

**Keywords:** CDK4/6, PET, abemaciclib, BBB, CNS

## Abstract

The synthesis, biochemical evaluation and radiosynthesis of a cyclin-dependent kinases 4 and 6 (CDK4/6) inhibitor and radioligand was performed. NT431, a newly synthesized 4-fluorobenzyl-abemaciclib, exhibited high potency to CDK4/6 and against four cancer cell lines with IC_50_ similar to that of the parent abemaciclib. We performed a two-step one-pot radiosynthesis to produce [^18^F]NT431 with good radiochemical yield (9.6 ± 3%, n = 3, decay uncorrected), high radiochemical purity (>95%), and high molar activity (>370 GBq/µmol (>10.0 Ci/µmol). In vitro autoradiography confirmed the specific binding of [^18^F]NT431 to CDK4/6 in brain tissues. Dynamic PET imaging supports that both [^18^F]NT431 and the parent abemaciclib crossed the BBB albeit with modest brain uptake. Therefore, we conclude that it is unlikely that NT431 or abemaciclib (FDA approved drug) can accumulate in the brain in sufficient concentrations to be potentially effective against breast cancer brain metastases or brain cancers. However, despite the modest BBB penetration, [^18^F]NT431 represents an important step towards the development and evaluation of a new generation of CDK4/6 inhibitors with superior BBB penetration for the treatment and visualization of CDK4/6 positive tumors in the CNS. Also, [^18^F]NT431 may have potential application in peripheral tumors such as breast cancer and other CDK4/6 positive tumors.

## 1. Introduction

Cyclin-dependent kinases 4 and 6 (CDK4/6) inhibitors suppress the dysregulation of the cell cycle by blocking the aberrantly accelerated cell cycle transition from the G1 to S phase [1,2]. This transition is regulated by a complex consisting of CDK4/6 and cyclin D, which phosphorylates retinoblastoma-associated proteins (pRb) and introduces E2F release [3,4,5]. CDK4/6 proteins have been reported to be overexpressed in a significant number of cancers such as head and neck cancer [6], non–small cell lung cancer [7], melanoma [8,9,10], and glioblastoma [11]. High levels of CDK4/6 overexpression have been observed in patients with luminal (i.e., estrogen receptor (ER)–positive) breast cancer, the most common subtype, representing approximately 75% of all breast cancers [12,13]. Moreover, overexpression of CDK4/6 appears to be an early step in cancer pathogenesis, as early as ductal carcinoma in situ (DCIS) and is maintained during progression to invasive locoregional and metastatic lesions [14,15,16]. Selective inhibitors of CDK4/6 indeed have been proven to be highly effective in large clinical trials of patients with advanced ER-positive breast cancer [17,18]. 

**Figure 1:**
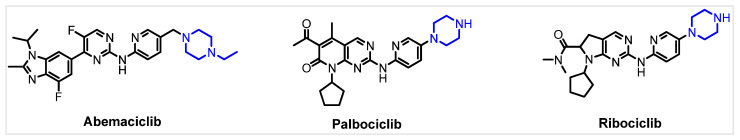
Chemical structures of the FDA approved CDK4/6 inhibitors: abemaciclib, palbociclib, and ribociclib.

Currently, abemaciclib (Verzenio), ribociclib (Kisqali), and palbociclib (Ibrance) (chemical structures are shown in Figure 1) are the three FDA-approved inhibitors of CDK4/6 used for treatment of ER-positive, HER2-negative advanced or metastatic breast cancer [19]. However, the utility of CDK4/6 for treatment of CNS tumors is limited due to poor penetration of the BBB [20]. It is likely that clinical trials [21,22] of CDK4/6 inhibitors in brain metastases did not meet their relevant endpoints because the drugs did not reach therapeutic concentrations sufficient for CDK4/6 inhibition in the CNS. Currently, non-invasive methods for measuring CDK4/6 expression in the brain are lacking. Therefore, there is a need for non-invasive tools to quantify CDK4/6 target engagement to ensure that CDK4/6 inhibitors reach concentrations in the brain sufficient to elicit pharmacological effects in tumor tissues and to provide information on response to treatment in real time. As such, positron emission tomography (PET) imaging with radiolabeled brain penetrant CDK4/6 inhibitors not only can measure and quantify target engagement but potentially will have clinical utility for the detection of CDK4/6 positive tumors in the CNS and how they respond to treatment. To this end, we developed the first BBB penetrant PET probe for the imaging of CDK4/6 in the CNS. 

## 2. Results

### 2.1. Chemical Synthesis and Characterization of Derivatives of NT431

**Figure 2:**
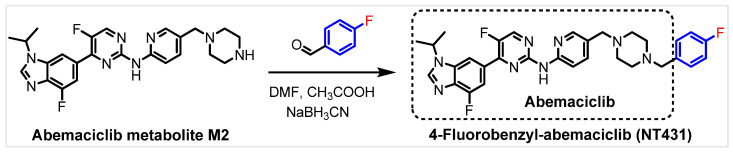
Chemical synthesis of 4-fluorobenzyl-abemaciclib (NT431), a minimally modified abemaciclib.

The straightforward synthesis of NT431 was accomplished by using reductive amination to couple the abemaciclib metabolite M2 with 4-fluorobenzaldehyde using acetic acid (CH_3_COOH) and sodium cyanoborohydride (NaBH_3_CN) as shown in Figure 2. NT431 was obtained with >90% chemical purity, and >50% chemical yield after purification by using flash chromatography and further purification using semi-prep HPLC. NT431 was characterized by using NMR spectroscopy (^1^H and ^19^F, Figure S1)) and the molecular weight was confirmed with high resolution mass spectroscopy (Figure S2). Purity was determined by using analytical HPLC (Figure S3). 

### 2.2. Biochemical Evaluation

The ability of NT431 to inhibit the CDK4/CyclinD3 and CDK6/CyclinD3 kinase activities was determined using the CDK4/CyclinD3 and the CDK6/CyclinD3 Kinase Assay Kits. The assay kits contain purified recombinant CDK4/CyclinD3 or CDK6/CyclinD3 kinases, kinase substrate, ATP, and kinase assay buffer. The experiments were conducted according to the protocol provided by the manufacturer, and abemaciclib was used as a positive control. 

**Figure 3:**
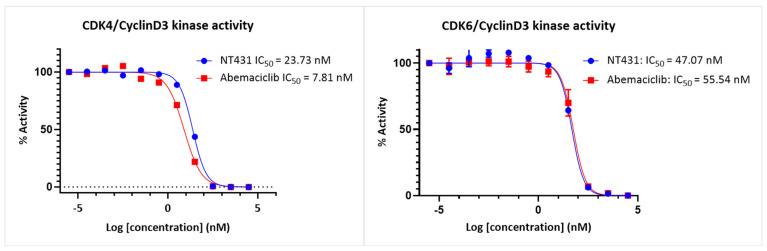
Inhibition of CDK4/CyclinD3 and CDK6/CyclinD3 kinase activities by NT431 and abemaciclib.

As shown in Figure 3, NT431 inhibited the CDK4/CyclinD3 and CDK6/CyclinD3 kinase activity with IC_50_ = 23.7 and 47.1 nM, respectively. The IC_50_ values for NT431 were comparable to that of the parent abemaciclib, which exhibited IC_50_ = 7.8 and 55.5 nM for CDK4/CyclinD3 and CDK6/CyclinD3, respectively. 

### 2.3. Evaluation of NT431 Cytotoxicity in Cancer Cells

We performed MTT assays to determine the cytotoxic effects of NT431 in cultured cancer cell lines MCF-7 (breast cancer), U87 and C6 (glioblastoma), and CC531 (rat colon carcinoma). All four cell lines were plated and treated with increasing concentrations of NT431 or abemaciclib for 48 h. The cell viability was assessed as described in Section 4. 

**Figure 4:**
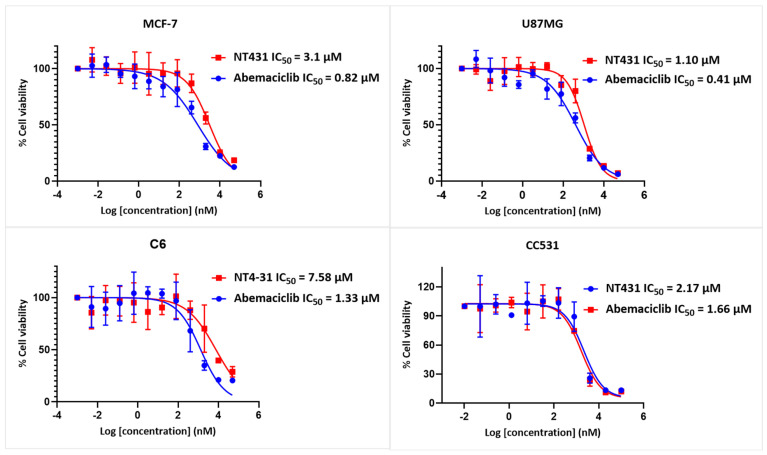
Cytotoxicity of NT431 evaluated in four cancer cell lines and compared side by side with abemaciclib.

The cell viability results in Figure 4 revealed that NT431 IC_50_ values of 3.1 µM for MCF-7 cells, 1.1 µM for U87MG cells, 7.6 µM for C6 cells, and 2.2 µM for CC531 cells. These results demonstrated that NT431 maintained the effective killing of various cancer cells, however, with lower efficacy compared to abemaciclib. 

These encouraging in vitro results with NT431 (Figure 3 and Figure 4) demonstrated high CDK4/6 inhibition in kinase activity assay combined with high efficacy against various cancer cell lines warranting the radiosynthesis of [^18^F]NT431 and the subsequent in vivo evaluation.

### 2.4. Radiosynthesis

**Figure 5:**
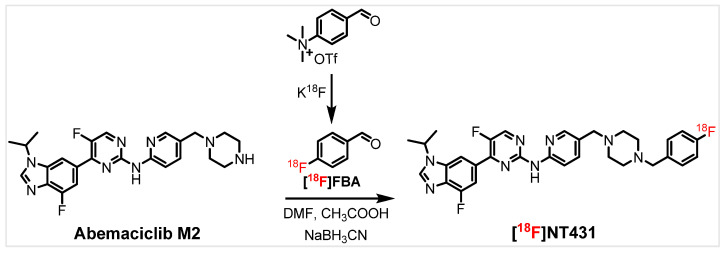
Radiochemical synthesis of the 4-[^18^F]fluorobenzyl-abemaciclib ([^18^F]NT431).

We performed a one-pot radiosynthesis of [^18^F]NT431 that was accomplished as shown in Figure 5. The 4-[^18^F]fluorobenzaldehyde (4-[^18^F]FBA) was produced as described previously [23]. Then, [^18^F]NT431 was produced by the subsequent reaction of the 4-[^18^F]FBA with abemaciclib metabolite M2 in the presence of acetic acid and NaBH_3_CN. The crude [^18^F]NT431 was purified by semi-prep HPLC and characterized by comparing the retention time of the radioactive tracer to the nonradioactive standard compound using analytical HPLC. [^18^F]NT431 was obtained with good radiochemical yield (24 ± 3.0%), high radiochemical purity (>95%), and high molar activity (>370 GBq/µmol, >10 Ci/µmol)). Overall, the one-pot radiochemical approach is straightforward and will likely facilitate the routine production of other 4-[^18^F]fluorobenzyl-modified CDK4/6 inhibitors. 

### 2.5. Formulation and In Situ Stability of [^18^F]NT431

[^18^F]NT431 was formulated in 20% ethanol and 80% solution of saline that contains sodium ascorbate (5 mg/mL). Radioactivity analysis with high performance liquid chromatography (radio-HPLC) demonstrated that [^18^F]NT431 in the formulated solution remained intact with >98% radiochemical purity for four hours post-formulation. 

### 2.6. In Vitro Autoradiography

**Figure 6:**
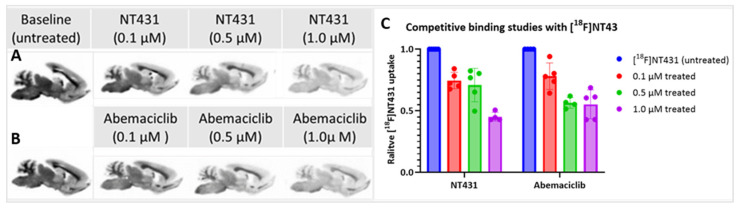
(**A**) Brain histology obtained with CDK4/6 antibodies. (**B**) In vitro autoradiography with [^18^F]NT431 at baseline and (**C**) Competitive blocking with NT431 and saturation with abemaciclib (1.0, 0.5, and 0.1 µM).

We performed in vitro autoradiography with [^18^F]NT431 in rat brain tissues using phosphor imaging as shown in Figure 6. In vitro autoradiography with [^18^F]NT431 was utilized at baseline and after competitive blocking to further confirm the specific binding of [^18^F]NT431 to CDK4/6 in the brain. This was established via competitive blocking with NT431 (self-blocking) or blocking with the parent FDA-approved abemaciclib at 1.0, 0.5, and 0.1 µM concentrations. The data in Figure 6C demonstrates a dose-dependent displacement of the [^18^F]NT431, thus indicating that the uptake of [^18^F]NT431 in the brain tissues was displaceable and specific to CDK4/6. These in vitro displacement studies with NT431 and abemaciclib were further confirmed by PET imaging in vivo as shown in Figure 6 below. It is also likely that higher concentrations (i.e., 5–10 µM) of the non-radioactive inhibitor will be needed to further delineate the specific binding and provide information about the non-specific binding. 

### 2.7. In Vivo PET and Preliminary Pharmacodynamic Imaging

**Figure 7:**
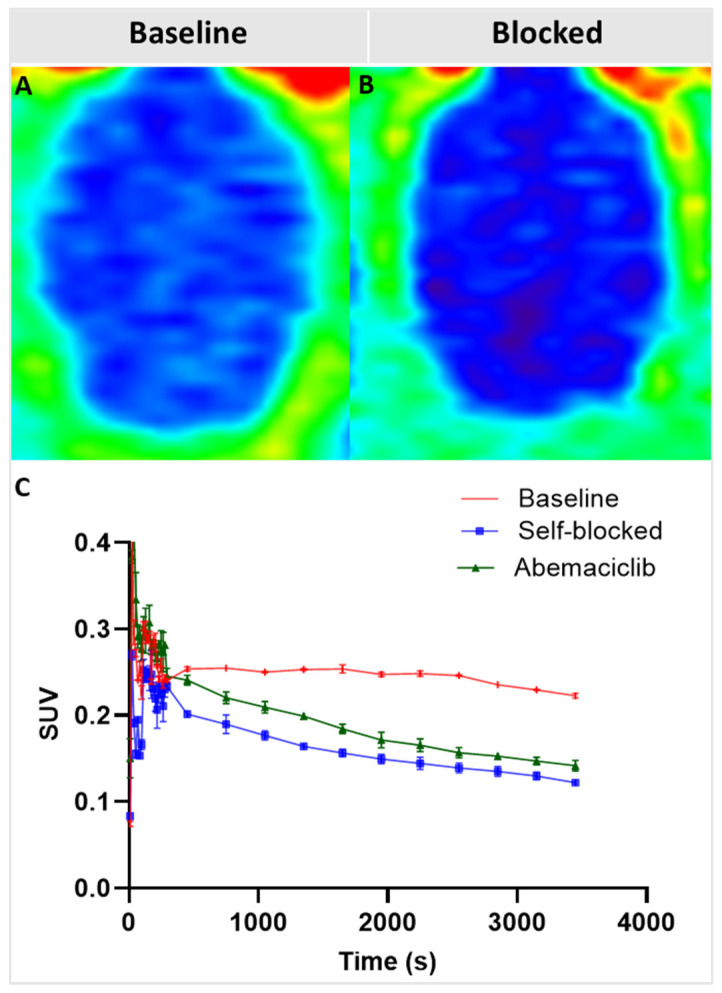
Representative coronal PET images (summed, 20–60 min) with [^18^F]NT431 in rat brain (**A**) at the baseline and (**B**) after self-blocking with 0.5 mg/kg of NT431. (**C**) Whole brain time–activity curves obtained from dynamic imaging over 60 min at baseline and after blocking with NT431 (self-blocking) or with abemaciclib (0.5 mg/kg each).

We performed in vivo PET imaging studies to determine the ability of [^18^F]NT431 to cross the blood brain barrier (BBB) in rat brain. Rat brain is relatively larger (compared to mouse brain) and allows for better tracer biodistribution and localization to quantify the background expression of CDK4/6 in the whole brain. As shown in Figure 7, [^18^F]NT431 crossed the BBB and accumulated in the brain in a specific manner as supported by competitive blocking studies by using non-radioactive excess of either NT431 or abemaciclib. 

The specific uptake of [^18^F]NT431 in the brain was confirmed in vivo through competitive blocking with the corresponding non-radioactive NT431 which led to a significant reduction in the radioactive signal time activity curve compared to the baseline (Figure 7B,C). Moreover, the data in Figure 7 demonstrates for the first time that abemaciclib can cross the BBB and enter the healthy brain, albeit with relatively low brain uptake. The specific binding of abemaciclib (excess) to CDK4/6 in the brain resulted in a significant reduction in the tracer time activity curve due to competitive binding to CDK4/6 by reducing the available binding sites for the radiotracer [^18^F]NT431. 

### 2.8. Radiometabolite Studies

We conducted radiometabolism studies in vivo to ensure that the PET imaging was performed while the parent radiotracer was mostly intact during the entire scan time (i.e., 60 min). The presence of radiometabolites in the brain can confound the PET data quantification by contributing to the CDK4/6 signal in the brain. The presence of radiometabolites in the brain can be attributed to brain radiometabolism (caused by metabolic enzymes in the brain itself) or blood radiometabolites capable of crossing the BBB. Therefore, we analyzed blood samples for the presence of radioactive metabolites to ensure that these metabolites did not cross the BBB in quantities sufficient to confound the PET signal. Typically, we expect the radiometabolites in blood to exhibit poor brain entry < 15% (changes < 15% cannot be detectable by PET [24,25]) and little or no interaction with CDK4/6 in the brain. Brain radiometabolites and radiometabolites in the blood that enter the brain can confound the PET data quantification since the PET detector cannot distinguish the parent radioactivity from the radiometabolites and instead detects them as one signal [26].

**Figure 8:**
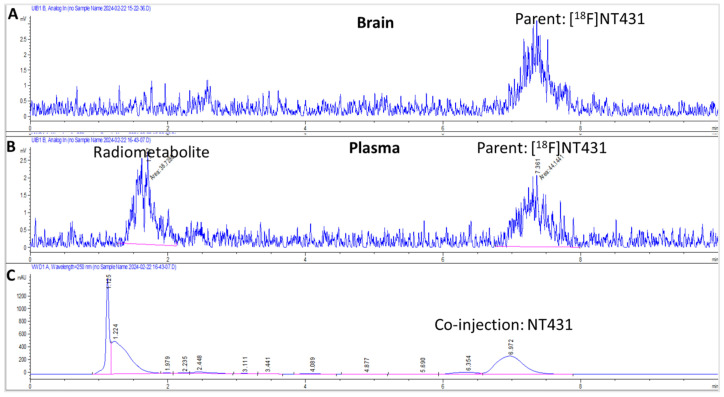
Analytical HPLC chromatograms of [^18^F]NT431 obtained from (**A**) brain homogenates; (**B**) plasma and (**C**) co-injection with authentic NT431. [^18^F]NT431 was eluted with 70% acetonitrile/ammonium acetate buffer (20.0 mM) at flow rate of 1.0 mL/min.

The extent of the radiometabolism of [^18^F]NT431 in rat brain and plasma at 60 min post-injection is shown in the radio-HPLC chromatogram (Figure 8). Analytical radio-HPLC revealed that the parent [^18^F]NT431 remained mostly intact in the brain during the entire scan time (Figure 8). The presence of polar radiometabolites in the brain homogenates was negligible, which indicates the absence of significant brain radiometabolism and that blood polar radiometabolites did not cross the BBB. Radio-HPLC analysis of the plasma at 60 min post-injection also showed a relatively higher fraction of the parent [^18^F]NT431 (54 ± 2%) and a single polar radiometabolite (46 ± 2%) (Figure 8). It is important to note that the recovery of radioactivity was ~73% and 42% from the brain homogenates and the plasma, respectively. The low radioactive recovery with acetonitrile is likely due to the high lipophilicity of [^18^F]NT431. Therefore, we cannot rule out the possibility that other radiometabolites may be present in the remaining precipitates. 

## 3. Discussion

CDK4/6 inhibitors have an immense potential for treatment of CDK4/6 positive CNS tumors such as glioblastoma and metastatic breast cancer [11,19]. However, the poor BBB penetration of approved CDK4/6 inhibitors likely explains their failure to control brain metastases in clinical trials [20,21,22]. Therefore, there is a need for non-invasive measures to quantitate the ability of CDK4/6 inhibitors to penetrate the BBB and accumulate in the brain in sufficient concentrations to elicit a pharmacological response. PET is a powerful non-invasive tool that can be used for such measurements and not only can provide information on the BBB penetration but can enable tumor visualization and provide information on response to treatment in real time. Currently, there is no tracer designed for PET imaging of CDK4/6 proteins in the CNS. [^18^F]CDKi, a modified palbociclib, was the only PET tracer that successfully delineated CDK4/6 in the MCF-7 breast cancer model [27]. However, CNS entry, specific uptake in the brain, and radiometabolism in the brain were not described. Other attempts with metal-based PET tracers entrapped in chelators extended from the piperazine moiety of CDK4/6 inhibitors led to abysmal tumor uptake [28,29,30,31]. The low lipophilicity associated with highly polar chelators and the relatively high molecular weights are likely factors that prevent metal-based tracers from entering the CNS. 

We selected abemaciclib among the approved CDK4/6-targeted drugs as a good starting point for radiolabeling because it was reported that abemaciclib was able to cross the BBB in sub µM concentrations to reach breast cancer brain metastases in human subjects [22]. Several reports in the literature and in patent applications demonstrate that the piperazine moiety (Figure 1, blue) of the FDA-approved molecules is susceptible to modification without compromising the high potency to CDK4/6 [27,32,33]. Therefore, we minimally modified abemaciclib using a functional group extended from the piperazine moiety to produce NT431, a 4-fluorobenzyl-abemaciclib. We then demonstrated that the new molecule NT431 maintained the high potency to CDK4/6 with an IC_50_ comparable to that of the parent abemaciclib. We then radiolabeled NT431 with [^18^F]fluoride to produce [^18^F]NT431 with good radiochemical yield, good radiochemical purity, and high molar activity. The two step one-pot radiochemistry is modular and can be extended to a wide range of molecules including other CDK4/6 inhibitors with piperazine extension. Next, we performed in vitro and in vivo studies to confirm BBB penetration and specific binding of NT431 to CDK4/6 in vitro and in vivo. [^18^F]NT431 displayed high radiometabolic stability in the brain with the parent remaining intact during the first 60 min of the scan. Moreover, no blood radiometabolites were detected in the brain as the blood radiometabolites that enter the brain can confound the PET image quantification. Moreover, the uptake of [^18^F]NT431 in the brain was susceptible to significant blocking (>50%) in vitro by the unlabeled version of the tracer (NT431) and by the CDK4/6 inhibitor abemaciclib. Also, as shown in Figure 7C, self-blocking with NT431 and competitive inhibition with abemaciclib were visualized in vivo as a decreased accumulation of the tracer in the brain. The time–activity curves obtained from [^18^F]NT431 at the baseline with images obtained from the tracer co-administered with NT431 or abemaciclib (0.5 mg/kg) resulted in a significant decrease in the time–activity curves in the whole brain (reduction in the SUV), which demonstrates that the binding/occupancy of [^18^F]NT431 to CDK4/6 in vivo is specific and displaceable. 

## 4. Materials and Methods

### 4.1. Reagents and Instruments

The starting material abemaciclib metabolite M2 (Cas No. 1231930-57-6) was obtained commercially (Muse Chem, Fairfield, NJ, USA). Abemaciclib (HY-16297A) was obtained from MedChemExpress (Monmouth Junction, NJ, USA). Solvents and other chemicals were obtained from MilliporeSigma (St. Louis, MO, USA) and were used as received. C18 light SPE cartridges were purchased from Waters (WAT023501, Milford, MA, USA).

Radiochemical purification of [^18^F]NT431 was performed using semipreparative HPLC (1260 series Agilent Technologies, Stuttgart, Germany) with an isocratic pump and built-in UV detector operated at 250 nm and a radioactivity detector with a single-channel analyzer (labLogic Chantilly, VA, USA) at a flow of 4 mL/min. We used C18 reverse-phase column (00G-4041-N0: Luna^®^ 5 µm, 250 × 10 mm (Phenomenex CA, USA)) to purify the tracer. Quality control analysis was performed using analytical HPLC (1260 series Agilent Technologies, Stuttgart, Germany) with a quaternary pump and a built-in variable wavelength detector, and a radioactivity detector with a single-channel analyzer (labLogic, Chantilly, VA, USA). The flow rate was 1.0 mL/min. A C18 column (00G-4041-N0: Luna^®^ 5 µm, 150 × 4.6 mm (Phenomenex, Torrance, CA, USA)) was used. 

An Agilent 1260HPLC/G6224A-TOF MS instrument was used for high resolution mass spectroscopy (HRMS) and a 400 MHz Bruker instrument was used for NMR spectroscopy. 

### 4.2. Chemical Synthesis

Chemical synthesis of 5-fluoro-4-(4-fluoro-1-isopropyl-1H-benzo[d]imidazol-6-yl)-N-(5-((4-(4-fluorobenzyl)piperazin-1-yl)methyl)pyridin-2-yl)pyrimidin-2-amine (NT431): Abemaciclib metabolite M2 (50 mg, 0.10 µmol) was dissolved in *N*,*N*-dimethylformamide (DMF, 1.0 mL) and treated with 4-fluorobenzaldehyde (50.0 µL, 0.40 µmol) followed by the addition of acetic acid (CH_3_COOH, 50.0 µL) and sodium cyanoborohydride (NaBH_3_CN, 200.0 mg, 3.20 µmol). The reaction mixture was heated at 70 °C overnight. The solvent was removed under reduced pressure, the residue was dissolved in dichloromethane, and the mixture was purified by flash chromatography (10% methanol:dichloromethane). The solvent was removed, and NT431 was collected as a yellowish powder. NT431 was obtained with >90% chemical purity and 50% chemical yield. ^1^H NMR (CDCl_3,_ 400 MHz) δ 8.46 (d, *J* = 4.0 Hz, 1H), 8.40 (d, *J* = 8.5 Hz, 1H), 8.30 (s, 1H), 8.26 (d, *J* = 1.8 Hz, 1H), 8.21 (s, 1H), 7.82 (d, *J* = 11.6 Hz, 1H), 7.70 (dd, *J*_1_ = 2.1, *J*_2_ = 8.5 Hz, 1H), 7.29 (t, *J* = 8.2 Hz, 1H, overlapped with the CDCl3 peak), 7.00 (t, *J* = 8.6 Hz, 1H), 4.76 (m, 1H), 3.50 (d, *J* = 9.7 Hz, 4H), 2.72 (s, 3H), 2.50 (bs, 8H), 1.73 (d, *J* = 7.0 Hz, 6H). ^19^F NMR (CDCl_3,_ 376.5 MHz), δ −116.00, −127.95, −148.24. HRMS: Calculated for C_32_H_34_F_3_N_8_ [M+H]^+^ 587.2853, Found: 587.2854.

### 4.3. Biochemical Evaluation and Cytotoxicity Studies

#### 4.3.1. CDK4/CyclinD3 and CDK6/CyclinD3 Kinase Activities

The CDK4/CyclinD3 Kinase Assay Kit (cat. # 79674) and the CDK6/CyclinD3 Kinase Assay Kit (cat. # 78395) were obtained from BPS Bioscience (San Diego, CA, USA). The inhibition of CDK4/CyclinD3 and CDK6/CyclinD3 kinase activities by NT431 was measured in the presence of increasing inhibitor concentrations using the CDK6/CyclinD3 and CDK6/CyclinD3 Kinase Assay Kits. The IC_50_ was determined graphically by measuring the luminescence using a microplate reader. 

#### 4.3.2. MTT Assay Protocol

Cells (MCF-7, U87MG or C6) were seeded on 96-well plates at a density of 5000 cells per well in 200 microliters of culture medium. Incubation overnight allowed for cell attachment. The culture medium from each well was removed, and 100 µL of the desired drug solutions (NT431 or abaculis) at the appropriate concentrations were added. The cells were then incubated for 48 h to allow the drugs to exert their effects on the cells. After the incubation period, the drug solutions were carefully removed from each well, and the cells were rinsed with phosphate-buffered saline (PBS) twice. Then, 100 microliters of MTT reagent (0.5 mg/mL) were added to each well. The plate was incubated for 3 h at 37 °C to allow the viable cells to convert MTT into formazan crystals. After the incubation, the MTT reagent was removed from each well, and 150 µL of dimethyl sulfoxide (DMSO) was added to dissolve the formazan crystals. The plate was gently shaken for 30 min to ensure complete dissolution. Once the formazan crystals were fully dissolved, the absorbance of the solution in each well was measured using a microplate absorbance reader at a wavelength of 570 nm. The data were analyzed by GraphPad Prism 9.

Radiosynthesis of 5-fluoro-4-(4-fluoro-1-isopropyl-1H-benzo[d]imidazol-6-yl)-N-(5-((4-(4-[^18^F]fluorobenzyl)piperazin-1-yl)methyl)pyridin-2-yl)pyrimidin-2-amine ([^18^F]NT431) was performed as follows.

The [18F]F- was trapped on a QMA cartridge (Waters, WAT023525) and eluted with kryptofix2.2.2 and K_2_CO_3_ and then dried similar to our previous reports [34,35]. The [18F]F- was trapped on a QMA cartridge and then eluted with 80:20% acetonitrile/water solution (1.0 mL) containing kryptofix2.2.2 (12.0 mg) and K_2_CO_3_ (1.0 mg) to a V-vial (Wheaton) to produce potassium [18F]fluoride/kryptofix2.2.2. The water/acetonitrile mixture was removed under a stream of Argon at 110 °C. Then acetonitrile (1.0 mL) was added to the residue, and the azeotropic drying under a stream of Argon at 110 °C was repeated two more times. 

The radiosynthesis of [^18^F]NT431 was performed in a two-step one-pot reaction with subsequent addition of the reactants at the appropriate time interval without radiochemical separation as described below. First, the radiosynthesis of 4-[^18^F]fluorobenzaldehyde (4-[^18^F]FBA) (produced in 7 min) was performed as previously reported [23]. Briefly, the precursor 4-formyl-*N*,*N*,*N*-trimethylbenzenaminium triflate (4.0–5.0 mg) in DMF (0.4 mL) was added to the dried [18F]fluoride/kryptofix2.2.2 and reacted at 130 °C for 7.0 min. Second, the key precursor abemaciclib metabolite M2 (5.0 mg) dissolved in acetic acid/DMSO (50.0 µL each) was added to the 4-[^18^F]FBA mixture followed by the addition of sodium cyanoborohydride (NaBH_4_CN, 10 mg) in DMSO (100 µL). The reaction mixture was heated at 120 °C for 7 min. The production of [^18^F]NT431 by reductive amination was confirmed through analytical radio-HPLC. The acetic acid was removed under a stream of argon followed by addition of water (3.0 mL). Solid phase separation (SPE) was performed by trapping [^18^F]NT431 on a short C-18 cartridge (Waters) that was washed with 10% acetonitrile/ammonium acetate solution (10.0 mL) followed by washing with water twice (2 × 10.0 mL). The trapped [^18^F]NT431 was then eluted with acetonitrile (1.0 mL), and ammonium acetate solution (NH_4_OAc: 20 mM) (0.5 mL) was added. [^18^F]NT431 was purified using semipreparative radio-HPLC system with a 1260 series pump (Agilent Technologies, Stuttgart, Germany) with a built-in UV detector operated at 250 nm and a radioactivity detector with a single-channel analyzer (labLogic). [^18^F]NT431 was eluted from the C18 reverse-phase column (00G-4041-N0: Luna^®^ 5 µm, 100 Å, 250 × 10 mm, Phenomenex) using 65% acetonitrile: 35% ammonium acetate buffer (NH_4_OAc: 20 mM) at flow rate of 4.0 mL/min. [^18^F]NT431 was collected at ~21.0 min post injection (Figure S3). Water (15.0 mL) was added to the collected solution, and the [^18^F]NT431 was trapped on a C18 light cartridge and eluted with ethanol (0.3 mL) and formulated for in vivo and in vitro studies. [^18^F]NT431 was formulated by the addition of a solution that contains 15% ethanol and 85% sodium ascorbate in water (5 mg/mL).

Generally, starting with ~5.55 GBq (~150 mCi) of [18F]F- led to a final dose of 0.54 ± 0.1 GBq (14.5 ± 3 mCi, 9.7 ± 3%, n = 3, decay uncorrected) of [^18^F]NT431 (on average, the radiosynthesis was completed in 90 min).

### 4.4. Molar Activity

The molar activity was determined from the area under the ultraviolet peak curve corresponding to the non-radioactive trace in the analytical HPLC chromatogram (Figure S4) against a calibration curve pre-prepared from the unlabeled reference standard. The molar activity of [^18^F]NT431 was >370.0 GBq/µmol (>10.0 Ci/µmol, n = 3).

### 4.5. PET Imaging Procedures in Animals

All studies were performed under a protocol approved by the Institutional Animal Care and Use Committee (IACUC) of Stony Brook University. In vivo microPET imaging studies were performed in Sprague Dawley (SD, Charles River) female rats (200–300 g, n = 2/experiment). The anesthetized SD rats were placed in the Inveon µPET (Siemens, Knoxville, TN, USA) in the supine position with the skull positioned in the center of the field of view. [^18^F]NT431 (~30.0 MBq (~0.8 mCi)/animal) was administered via the tail-vein injection in a total volume 0.5–1.2 mL. Dynamic PET images were acquired for 60 min. Images were reconstructed with attenuation correction using an ordered subset expectation maximization (OSEM2D) algorithm with 16 subsets and 4 iterations. PET image analysis was performed using the AMIDE software similar to our previous report [36]. The time–activity curves (TAC) were generated manually by drawing the region of interest (ROI: whole brain) followed by plotting the radioactivity/cc vs time. Levels of accumulation of the radiotracer in tissues were expressed as standard uptake values (SUV) that were calculated for the regions of interest (ROI) using the AMIDE software. GraphPad Prism 10.2.0 (Graph Pad Software La Jolla, CA, USA) was used to blot the data.

### 4.6. In Vitro Autoradiography

The female rats (n= 3) were sacrificed, and their brains were excised and fixed in 4% PFA overnight, and then they were embedded into paraffin blocks. Eight 8 μm thick slices were obtained successively from each rat brain for further analysis. The slices were then deparaffinized in xylene three times for 5 min each, followed by rehydration with 100% ethanol, 95% ethanol, 70% ethanol, and then rinsed in R.O water for 30 min.

Next, the slides were preincubated in the binding buffer (50 mM Tris base, 2 mM MgCl_2_, pH 7.4) and 0.1% BSA + 20% ethanol for 2 h. The experiments were divided into seven groups: one group served as the control and was soaked in 25 mL of 0.13 MBq (3.5 μCi)/mL of ^18^F-labeled tracers; the second group was soaked in 25 mL of 0.13 MBq (3.5 μCi)/mL of [^18^F]NT431 + 0.1 μM NT431; the third group was soaked in 25 mL of 0.13 MBq (3.5 μCi)/mL of [^18^F]NT431 + 0.5 μM NT431; the fourth group was soaked in 25 mL of 0.13 MBq (3.5 μCi)/mL of [^18^F]NT431 + 1.0 μM NT431; the fifth group was soaked in 25 mL of 0.13 MBq (3.5 μCi)/mL of [^18^F]NT431 + 0.1 μM abemaciclib; the sixth group was soaked in 25 mL of 0.13 MBq (3.5 μCi)/mL of [^18^F]NT431 + 0.5 μM abemaciclib; and the seventh group was soaked in 25 mL of 0.13 MBq (3.5 μCi)/mL of [^18^F]NT431 + 1.0 μM abemaciclib. All the slices were incubated at room temperature for 150 min. Subsequently, slides were washed twice for 10 min in ice-cold binding buffer and dipped in ice-cold water before being dried. The slides were placed onto the storage phosphor screen in the dark overnight, and the images were collected using Typhoon FLA7000. Quantity image 5.2 was used to quantify radiation energy. The data were statistically analyzed using Prism 9 with Ordinary one-way ANOVA-Multiple comparisons.

### 4.7. Radiometabolism of [^18^F]NT431 

The extent of radiometabolism of [^18^F]NT431 was determined in rat brain homogenates and plasma according to the following protocol. SD rats (n = 2) were injected via the tail-vein injection with [^18^F]NT431 (52.0 MBq, 1.4 mCi/animal) in a total volume of 1.2 mL of the formulated solution. At one hour post-injection the blood (3 mL) was withdrawn from the heart while the rats were under terminal anesthesia with isoflurane. Then the rats were euthanized, and the brains were excised. The radioactivity in the brain and the blood was counted using a gamma counter (COBRAII D5003). 

The rat brains were homogenized in acetonitrile (2.0 mL) followed by centrifugation (Eppendorf 5453) at 12,000 rpm for 3 min, then the supernatant was collected, and the radioactivity was counted using a gamma counter. The recovery of radioactivity from the brain homogenates was ~73% which is consistent with other lipophilic PET tracers produced in our laboratory. The solution was passed through 0.22 µm filter, and then a sample (200 µL) was injected into the analytical HPLC for analysis.

To obtain protein-free plasma, the collected blood samples were centrifuged (Eppendorf 5702) at 4000 rpm for 5 min. Acetonitrile (2.0 mL) was then added, and the mixture was centrifuged (Eppendorf 5453) at 12,000 rpm for 3 min. The supernatant was collected, and the radioactivity was measured by using a gamma counter. The recovery was ~42%. The solution was passed through a 0.22 µm filter, and a sample (200 µL) from the resulting filtrates was injected into the analytical HPLC for analysis. The extent of radiometabolism of [^18^F]NT431 in the brain and the blood was determined by integrating the radioactive peaks compared to the parent [^18^F]NT431 (Figure 8). 

## 5. Conclusions

We developed [^18^F]NT431 as the first brain-penetrant CDK4/6 probe for PET imaging studies of CDK4/6 expression in the CNS in vivo. We demonstrated in vitro and in vivo that the uptake of [^18^F]NT431 in the brain is specific and displaceable through competitive blocking with excess NT431 or abemaciclib. Our PET imaging results demonstrated that [^18^F]NT431 and subsequently the parent abemaciclib cross the BBB, however, unlikely at sufficient concentrations to be pharmacologically effective against breast cancer brain metastases or brain cancers. Despite the modest BBB uptake, [^18^F]NT431 represents an important step towards the future evaluation and development of CDK4/6 inhibitors or radioligands with superior BBB penetration for treatment and visualization of CDK4/6 positive cancers in the CNS. Also, [^18^F]NT431 may have potential applications in peripheral tumors such as breast cancer and breast cancer metastasis. 

## Data Availability

All data generated or analyzed during this study are included in this published article and its Supplementary Materials.

## Supplementary Materials

The following supporting information can be downloaded at: https://www.mdpi.com/article/10.3390/ijms25136870/s1.
