# Supplementary material for: Development of a Radiolabeled Cyclin-Dependent Kinases 4 and 6 (CDK4/6) Inhibitor for Brain and Cancer PET Imaging
Source: Int J Mol Sci. 2024 Jun 22;25(13):6870. doi: 10.3390/ijms25136870 (PMC11241330; doi:10.3390/ijms25136870)
Supplement: Supplementary file 1 [file ijms-25-06870-s001.zip › ijms-3032835-supplementary.pdf]

Development of a Radiolabeled Cyclin-dependent Kinases 4 and 6 (CDK4/6) Inhibitor for Brain and Cancer PET Imaging

Chun-Han Huang[1,2,3], Palwasha Khan[1,2], Sulan Xu[1,2], Jules Cohen[1,4], Georgios V. Georgakis[1,5], Nashaat Turkman*[1,2,3]

[1] Stony Brook Cancer Center, Stony Brook, Long Island, 11794, NY, USA

[2] Department of Radiology, School of Medicine, Stony Brook University, Long Island, 11794, NY, USA

[3] Department of Biomedical Engineering, Stony Brook University, Long Island, 11794, NY, USA

[4] Department of Medicine, School of Medicine, Stony Brook University, Long Island, 11794, NY, USA

[5] Department of Surgery, School of Medicine, Stony Brook University, Long Island, 11794, NY, USA

*Corresponding author:

Nashaat Turkman, email: Nashaat.Turkman@stonybrookmedicine.edu

Department of Radiology, School of Medicine, Stony Brook University, 100 Nicolls Road, Stony Brook, 11794, NY, USA.

Tel: +01- 631-444-7938

Table of Contents **Page**

^{1}H NMR ($CDCl_3$, 400 MHz), and B) ^{19}F NMR ($CDCl_3$, 376.5 MHz) spectrums for NT431 2

High resolution spectroscopy for NT431 3

Semi-prep and analytical radio-HPLC chromatograms of [^{18}F]NT431 4

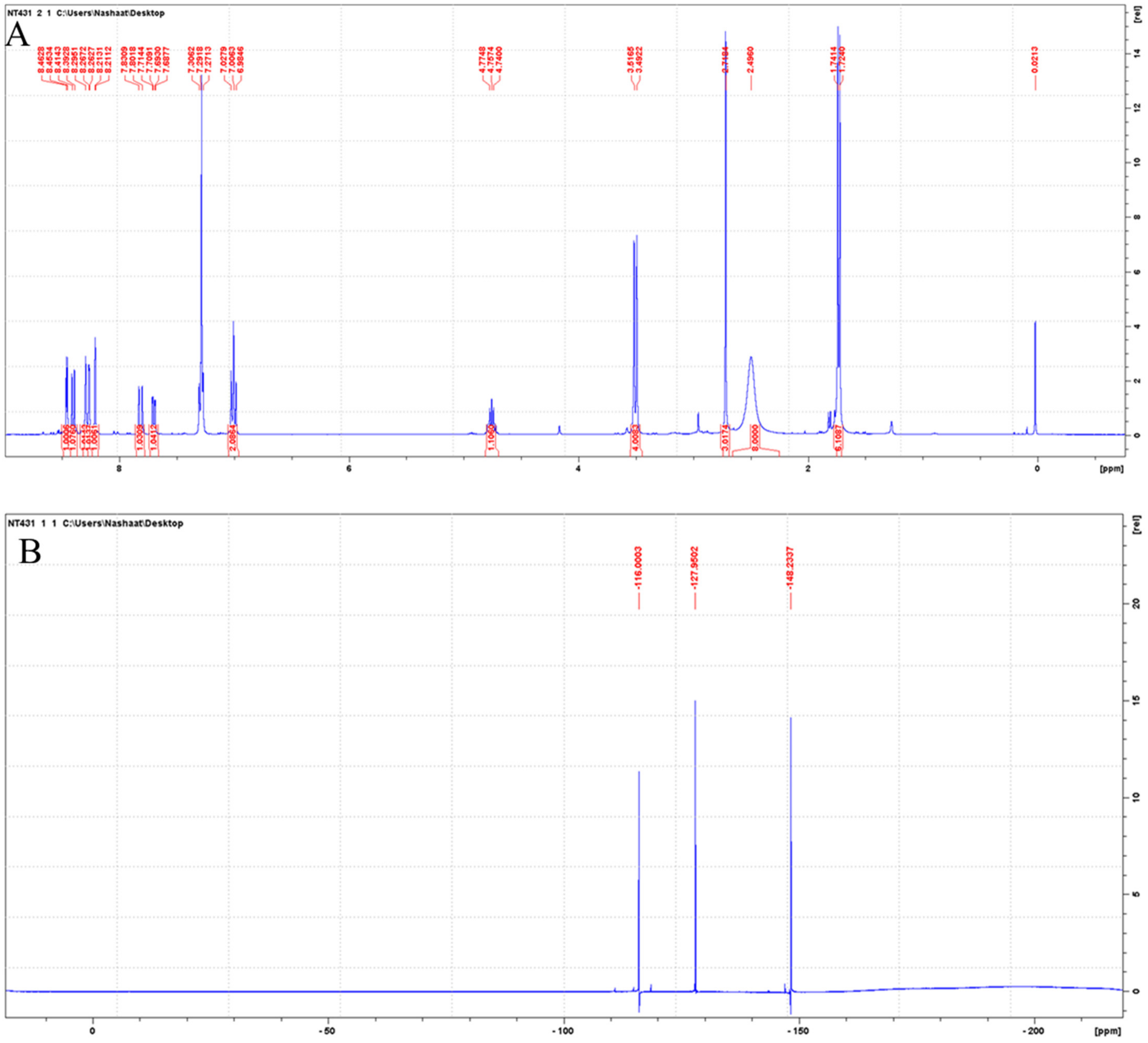

Figure S1 A) ^{1}H NMR ($CDCl_3$, 400 MHz) and B) ^{19}F NMR ($CDCl_3$, 376.5 MHz) spectroscopic characterization for NT431 was performed using 400 MHz Bruker instrument.

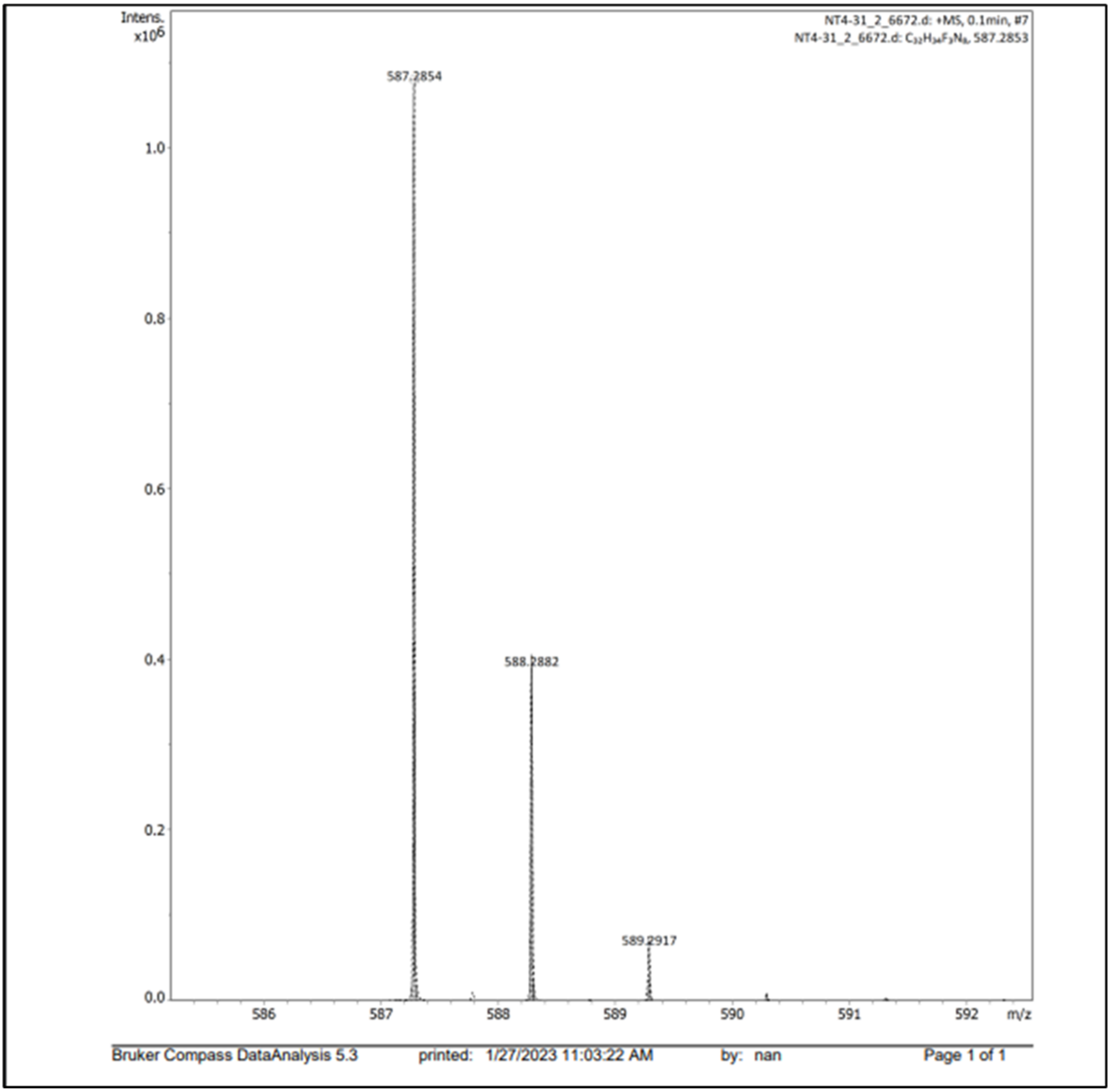

Figure S2 High resolution mass spectroscopy (HRMS) for NT431 was performed using Agilent 1260HPLC/G6224A-TOF MS. Calculated for $C_{32}H_{34}F_3N_8$ $[M+H]^+$ 587.2853, Found: 587.2854.

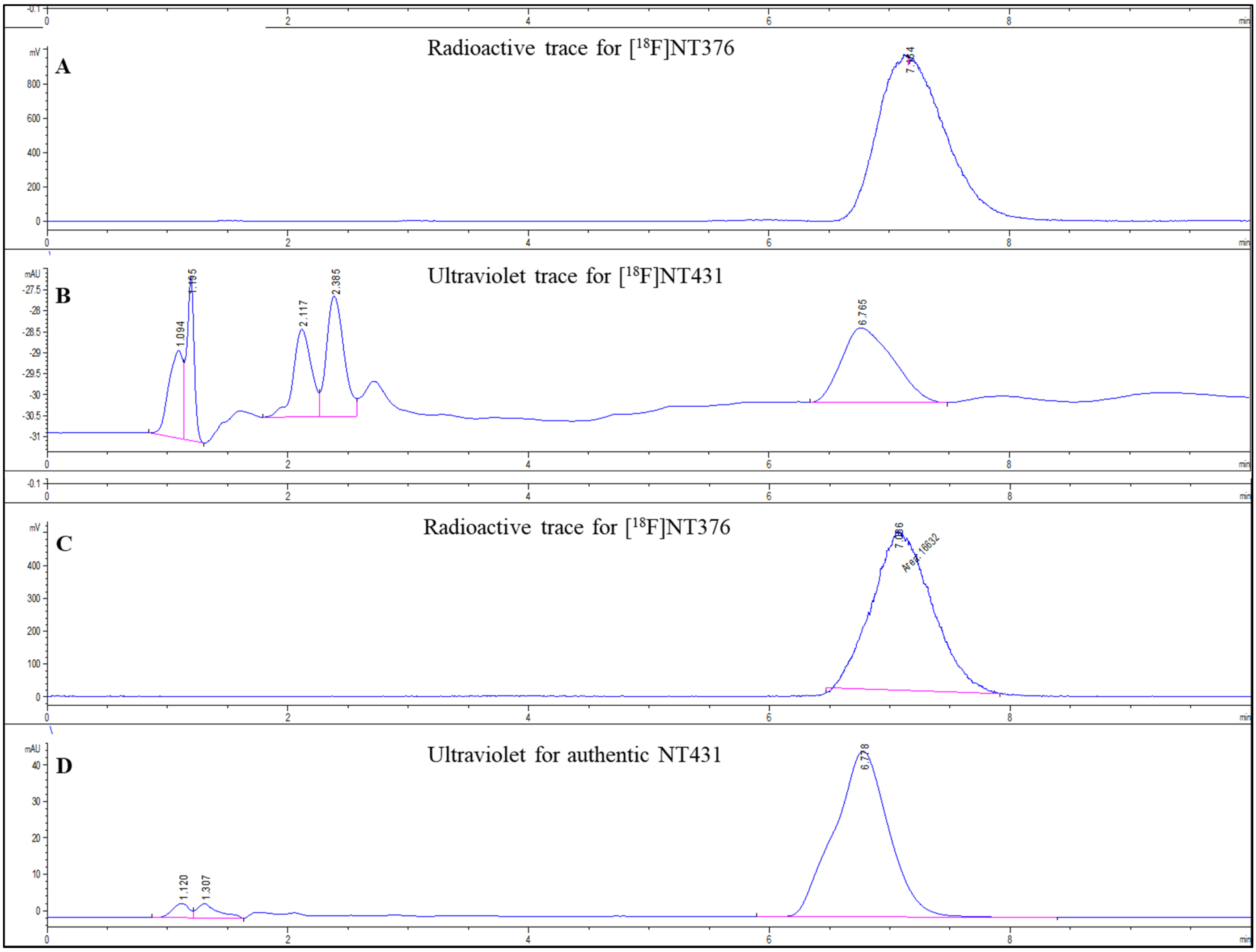

Figure S3 Analytical high performance liquid chromatography (HPLC) qaulity control for **[^{18}F]NT431** (a) radioactive peak of **[^{18}F]NT431** detected with radioactivity detector: radiochemical purity >96%, (b) **[^{18}F]NT431** associated non-radioactive mass detected with ultraviolet detector. (C) radioactive peak of **[^{18}F]NT431** co-injected with D) authentic **NT431** detected with ultraviolet detector. Samples were eluted with 70% acetonitrile/30% ammonium acetate buffer (20.0 mM) at a flow rate of 1.0 mL/min.

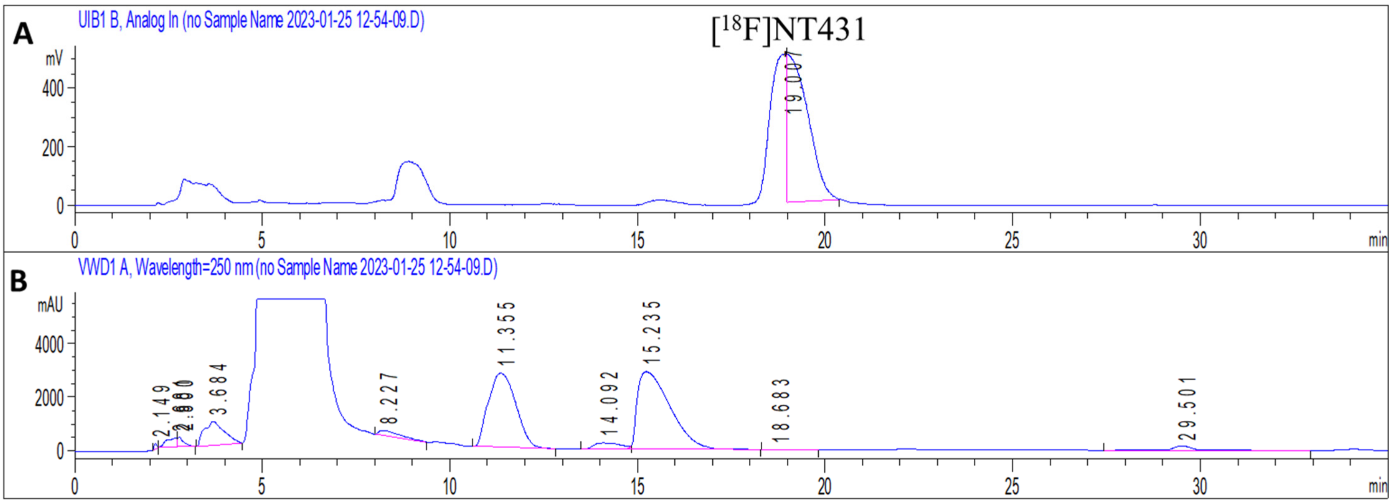

Figure S4 Semi-preparative HPLC purification of [^{18}F]NT431 (a) radioactive peak of [^{18}F]NT431 in crude mixture detected with radioactivity detector, (b) non-radioactive crude was detected with ultraviolet detector. The purification was performed using 65% acetonitrile: 35% ammonium acetate buffer (NH_4OAc: 20 mM) at flow rate of 4.0 mL/minute.
